# Structural and electronic properties of germanene/MoS_2_ monolayer and silicene/MoS_2_ monolayer superlattices

**DOI:** 10.1186/1556-276X-9-110

**Published:** 2014-03-08

**Authors:** Xiaodan Li, Shunqing Wu, Sen Zhou, Zizhong Zhu

**Affiliations:** 1 Department of Physics, Xiamen University, Xiamen 361005, China; 2 Institute of Theoretical Physics and Astrophysics, Xiamen University, Xiamen 361005, China; 3 State Key Laboratory of Theoretical Physics, Institute of Theoretical Physics, Chinese Academy of Sciences, Beijing 100864, China

**Keywords:** Superlattice, MoS_2_ monolayer, Germanene, Silicene

## Abstract

Superlattice provides a new approach to enrich the class of materials with novel properties. Here, we report the structural and electronic properties of superlattices made with alternate stacking of two-dimensional hexagonal germanene (or silicene) and a MoS_2_ monolayer using the first principles approach. The results are compared with those of graphene/MoS_2_ superlattice. The distortions of the geometry of germanene, silicene, and MoS_2_ layers due to the formation of the superlattices are all relatively small, resulting from the relatively weak interactions between the stacking layers. Our results show that both the germanene/MoS_2_ and silicene/MoS_2_ superlattices are manifestly metallic, with the linear bands around the Dirac points of the pristine germanene and silicene seem to be preserved. However, small band gaps are opened up at the Dirac points for both the superlattices due to the symmetry breaking in the germanene and silicene layers caused by the introduction of the MoS_2_ sheets. Moreover, charge transfer happened mainly within the germanene (or silicene) and the MoS_2_ layers (intra-layer transfer), as well as some part of the intermediate regions between the germanene (or silicene) and the MoS_2_ layers (inter-layer transfer), suggesting more than just the van der Waals interactions between the stacking sheets in the superlattices.

## Background

In the past decade, the hybrid systems consisting of graphene and various two-dimensional (2D) materials have been studied extensively both experimentally and theoretically [1-6]. It has long been known that the thermal, optical, and electrical transport properties of graphene-based hybrids usually exhibit significant deviations from their bulk counterparts, resulting from the combination of controlled variations in the composition and thickness of the layers [6,7]. Moreover, the use of 2D materials could be advantageous for a wide range of applications in nanotechnology [8-13] and memory technology [14-16]. Among those hybrid systems, the superlattices are considered as one of the most promising nanoscale engineered material systems for their possible applications in fields such as high figure of merit thermoelectrics, microelectronics, and optoelectronics [17-19]. While the research interest in graphene-based superlattices is growing rapidly, people have started to question whether the graphene could be replaced by its close relatives, such as 2D hexagonal crystals of Si and Ge, so called silicene and germanene, respectively. Silicene and germanene are also zero-gap semiconductors with massless fermion charge carriers since their *π* and π* bands are also linear at the Fermi level [20]. Systems involving silicene and germanene may also be very important for their possible use in future nanoelectronic devices, since the integration of germanene and silicene into current Si-based nanoelectronics would be more likely favored over graphene, which is vulnerable to perturbations from its supporting substrate, owing to its one-atom thickness.

Germanene (or silicene), the counterpart of graphene, is predicted to have a geometry with low-buckled honeycomb structure for its most stable structures unlike the planar one of graphene [20-22]. The similarity among germanene, silicene, and graphene arises from the fact that Ge, Si, and C belong to the same group in the periodic table of elements, that is, they have similar electronic configurations. However, Ge and Si have larger ionic radius, which promotes *sp*^3^ hybridization, while *sp*^2^ hybridization is energetically more favorable for C atoms. As a result, in 2D atomic layers of Si and Ge atoms, the bonding is formed by mixed *sp*^2^ and *sp*^3^ hybridization. Therefore, the stable germanene and silicene are slightly buckled, with one of the two sublattices of the honeycomb lattice being displaced vertically with respect to the other. In fact, interesting studies have already been performed in the superlattices with the involvement of germanium or/and silicon layers recently. For example, the thermal conductivities of Si/SiGe and Si/Ge superlattice systems are studied [23-25], showing that either in the *cross*- or *in-plane* directions, the systems exhibit reduced thermal conductivities compared to the bulk phases of the layer constituents, which improved the performance of thermoelectric device. It is also found that in the ZnSe/Si and ZnSe/Ge superlattices [26], the fundamental energy gaps increase with the decreasing superlattice period and that the silicon or/and germanium layer plays an important role in determining the fundamental energy gap of the superlattices due to the spatial quantum confinement effect. Hence, the studies of these hybrid materials should be important for designing promising nanotechnology devices.

In the present work, the structural and electronic properties of superlattices made with alternate stacking of germanene and silicene layers with MoS_2_ monolayer (labeled as Ger/MoS_2_ and Sil/MoS_2_, respectively) are systematically investigated by using a density functional theory calculation with the van der Waals (vdW) correction. In addition, we compare the results of Ger/MoS_2_ and Sil/MoS_2_ superlattices with the graphene/MoS_2_ superlattice [6] to understand the properties concerning the chemical trend with the group IV atoms C, Si, and Ge in the superlattices. Our results show that Ger/MoS_2_ and Sil/MoS_2_ consist of conducting germanene and silicene layers and almost-insulating MoS_2_ layers. Moreover, small band gaps open up at the *K* point of the Brillouin zone (BZ), due to the symmetry breaking of the germanene and silicene layers which is caused by the introduction of the MoS_2_ layers. Localized charge distributions emerged between Ge-Ge or Si-Si atoms and their nearest neighboring S atoms, which is different from the graphene/MoS_2_ superlattice, where a small amount of charge transfers from the graphene layer to the MoS_2_ sheet [6]. The contour plots for the charge redistributions suggest that the charge transfer between some parts of the intermediate regions between the germanene/silicene and the MoS_2_ layers is obvious, suggesting much more than just the van der Waals interactions between the stacking sheets in the superlattices.

## Methods

The present calculations are based on the density functional theory (DFT) and the projector-augmented wave (PAW) representations [27] as implemented in the Vienna *Ab Initio* Simulation Package (VASP) [28,29]. The exchange-correlation interaction is treated with the generalized gradient approximation (GGA) which is parameterized by Perdew-Burke-Ernzerhof formula (PBE) [30]. The standard DFT, where local or semilocal functionals lack the necessary ingredients to describe the nonlocal effects, has shown to dramatically underestimate the band gaps of various systems. In order to have a better description of the band gap, corrections should be added to the current DFT approximations [31,32]. On the other hand, as is well known, the popular density functionals are unable to describe correctly the vdW interactions resulting from dynamical correlations between fluctuating charge distributions [33]. Thus, to improve the description of the van der Waals interactions which might play an important role in the present layered superlattices, we included the vdW correction to the GGA calculations by using the PBE-D2 method [34]. The wave functions are expanded in plane waves up to a kinetic energy cutoff of 420 eV. Brillouin zone integrations are approximated by using the special ***k***-point sampling of Monkhorst-Pack scheme [35] with a Γ-centered 5 × 5 × 3 grid. The cell parameters and the atomic coordinates of the superlattice models are fully relaxed until the force on each atom is less than 0.01 eV/Å.

## Results and discussions

**Figure 1:**
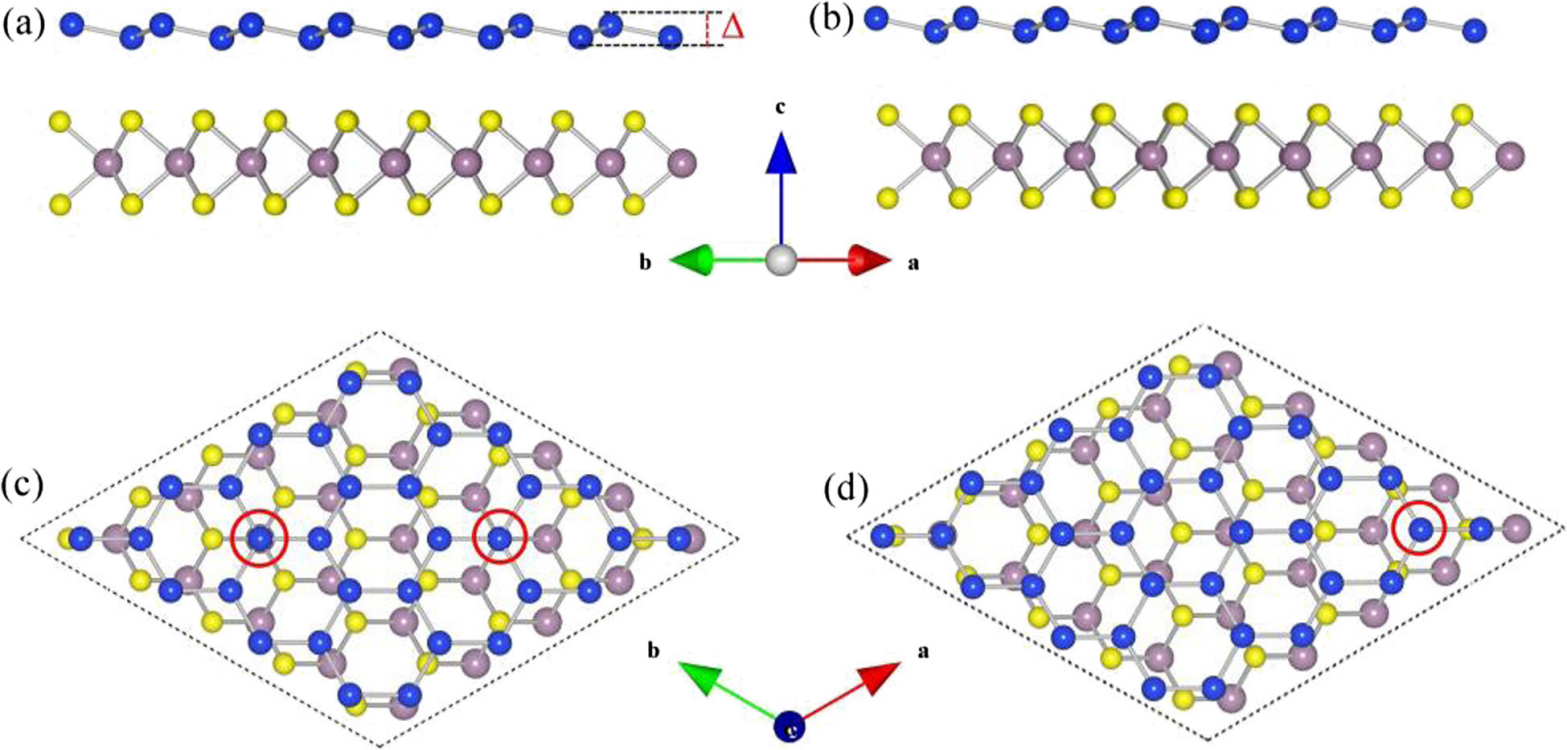
**Side and top views of the two arrangements of germanene/silicene on MoS**_**2**_**. (a, c)** Top site configuration; **(b, d)** hollow site configuration. Ge/Si, Mo, and S atoms are represented by blue, purple, and yellow balls, respectively. The unit cells are shown by dashed lines.

For the free-standing low-buckled germanene and silicene, the calculated lattice constants are 4.013 and 3.847 Å, respectively, which agree well with the reported values of 4.061 and 3.867 Å for germanene and silicene, respectively [36]. Our optimized lattice constant for a MoS_2_ monolayer is 3.188 Å, which is the same as the previous calculated values by PBE calculations [37]. Although the lattice constants of germanene/silicene and MoS_2_ monolayer are quite different, all of them do share the same primitive cell of hexagonal structure. For establishing the calculation models for Ger/MoS_2_ and Sil/MoS_2_ superlattices and to minimize the lattice mismatch between the stacking sheets, we have employed supercells consisting of 4 × 4 unit cells of germanene (and silicene) and 5 × 5 unit cells of MoS_2_ monolayer in the *x*-*y* plane. Thus, we have 4*a*(gemanene) = 16.052 Å, 4*a*(silicene) = 15.388 Å, and 5*a*(MoS_2_ monolayer) = 15.940 Å, which lead to a lattice mismatch of around 0.70% between the germanene and MoS_2_ layers and 3.46% between the silicene and MoS_2_ layers. Compared with the hybrid systems investigated previously [38-42], the present lattice mismatch values are very small. In the calculations, first, the lattice constant of germanene/silicene (4*a*_ger/sil_) was set to match to that $\left(5a_{{\mathrm{MoS}}_{2}}\right)$ of the MoS_2_ monolayer in the supercell. The supercells are then fully relaxed for both the lattice constants and the atomic geometry. The mismatch will finally disappear, leading to the commensurate systems. The superlattices we introduced in this work, by hybridizing germanene or silicene with MoS_2_ monolayer, are shown in Figure 1. The supercells consist of alternate stacking of one germanene or silicene sheet and one MoS_2_ monolayer, with 32 Ge or Si atoms, 25 Mo, and 50 S atoms per supercell. For a single Ge or Si atom adsorbed on a MoS_2_ monolayer, there are three possible adsorption sites, i.e., the top site directly above a Mo atom, the top site directly above a S atom, and the hollow site above the center of a Mo-S hexagon. For the Ger/MoS_2_ and Sil/MoS_2_ superlattices, we consider two possible representative arrangements of germanene/silicene on the MoS_2_ monolayer: (i) one Ge or Si atom in the supercell (4 × 4 unit cell) was set to sit directly on top of one Mo/S atom (the positions of all the other Ge or Si atoms will then be determined). In this way, there will be one Ge or Si atom in the supercell sitting on top of a S/Mo atom, too; see Figure 1c. (ii) One Ge or Si atom in the supercell was set to sit on the hollow site above the center of a hexagon of MoS_2_, as shown in Figure 1d. From the present calculations, it is found that the binding energy differences between the above models of superlattices are very small (about 1 to 2 meV), which indicates that the energy of superlattice is not sensitive to the stacking of the atomic layers. Thus, in this paper, we show only the results of the configuration with one Ge or Si atom on top of the Mo or S atom. In all the stacking types, the 2D characteristics of the superlattice structures are kept, e.g., hexagonal atomic networks are seen in both Figure 1c,d which shows the fully optimized geometric structures of the supercells. Actually, the changes of the superlattice structures are quite small by atomic relaxations. The calculated lattice constants of Ger/MoS_2_ and Sil/MoS_2_ superlattices are 15.976 and 15.736 Å, respectively. In the Ger/MoS_2_ superlattice, the germanene layers are compressed by 0.47% (from 4.013 to 3.994 Å) as compared to the corresponding isolated germanene, while the MoS_2_ layers are expanded by 0.22% (from 3.188 to 3.195 Å) as compared to the free-standing MoS_2_ monolayer. On the other hand, in the case of Sil/MoS_2_ superlattice, the silicene layers in the superlattice are expanded by 2.26% (from 3.847 to 3.934 Å), while the MoS_2_ layers in the supercell are reduced by 1.29% (from 3.188 to 3.147 Å) (see Table 1).

**Table 1:** Binding energies, geometries, supercell lattice constants, averaged bond lengths, sheet thicknesses, and buckling of superlattices

**System**	***E***_**b**_**(per Ge/Si)**	***E***_**b**_**(per MoS**_**2**_**)**	***a = b***	***c***	***d***_**Mo-S**_	***d***_**Ge-Ge**_**/*d***_**Si-Si**_	***h***_**S-S**_	***Δ***_**Ge**_	***Δ***_**Si**_
	**(eV)**	**(eV)**	**(Å)**	**(Å)**	**(Å)**	**(Å)**	**(Å)**	**(Å)**	**(Å)**
Ger/MoS_2_	0.277	0.354	15.976	9.778	2.410 to 2.430	2.420 to 2.440	3.129	0.782	
Sil/MoS_2_	0.195	0.250	15.736	9.926	2.400 to 2.410	2.320 to 2.330	3.176		0.496
Germanene			16.052			2.422		0.706	
Silicene			15.388			2.270			0.468
MoS_2_ monolayer			15.940		2.413		3.118		

Theoretical geometries of the isolated germanene, silicene, and MoS_2_ monolayer are also listed. *E*_b_, binding energies (per Ge/Si atom and per MoS_2_); *a*, *b*, and *c*, supercell lattice constants; *d*_Mo-S_, *d*_Ge-Ge_, and *d*_Si-Si_, averaged Mo-S and Ge-Ge/Si-Si bond lengths; *h*_S-S_, sheet thicknesses of MoS_2_; *Δ*_Ge_ and *Δ*_Si_, amplitude of buckling of the germanene and silicene in the superlattices.

The averaged Mo-S bond lengths of the superlattices are calculated to be all around 2.400 Å (see Table 1). The averaged Ge-Ge/Si-Si bond lengths (*d*_Ge-Ge_/*d*_Si-Si_) in the relaxed superlattices are all around 2.400/2.300 Å, which are close to those in the free-standing germanene/silicene sheets (2.422/2.270 Å). Although the atomic bond lengths in the stacking planes are almost the same for Ger/MoS_2_ and Sil/MoS_2_ superlattices, the interlayer distances (*d*) exhibit relatively larger deviations (but still close to each other; see Table 1). A shorter interlayer distance *d* is found in the Ger/MoS_2_ system, indicating that the Ge-MoS_2_ interaction is stronger than the Si-MoS_2_ interaction in the Sil/MoS_2_ system. The Ge-S and Si-S atomic distances in the Ger/MoS_2_ and Sil/MoS_2_ superlattices are 2.934 and 3.176 Å, respectively, where both values are shorter than 3.360 Å in the graphene/MoS_2_ superlattice [6]. Such decreases of interlayer distances indicate the enhancement of interlayer interactions in the Ger/MoS_2_ and Sil/MoS_2_ superlattices as compared to the graphene/MoS_2_ one. This can also explain why the amplitude of buckling (*Δ*) in the germanene/silicene layers of the superlattices become larger as compared to the free-standing germanene/silicene, i.e., *Δ* going from 0.706 to 0.782 Å in the germanene layers and from 0.468 to 0.496 Å in the silicene layers. The Ge-S and Si-S atomic distances in the Ger/MoS_2_ and Sil/MoS_2_ superlattices (2.934 and 3.176 Å) are much larger than 2.240 and 2.130 Å, the sum of the covalent atomic radius of Ge-S and Si-S atoms (the covalent radius is 1.220/1.110 Å for germanium/silicon and 1.020 Å for sulfur), which suggests that the interlayer bonding in the superlattices is not a covalent one.

To discuss the relative stabilities of the superlattices, the binding energy between the stacking sheets in the superlattice is defined as $E_{b}=-\left[E_{\sup\mathrm{ercell}}-\left(E_{{\mathrm{MoS}}_{2}}+E_{\mathrm{Ger}/\mathrm{Sil}}\right)\right]/N$, where *E*_supercell_ is the total energy of the supercell, and $E_{{\mathrm{MoS}}_{2}}$ and *E*_Ger/Sil_ are the total energies of a free-standing MoS_2_ monolayer and an isolated germanene/silicene sheet, respectively. When *N = N*(Ge/Si) = 32, the number of Ge/Si atoms in the supercell, *E*_b_ is then the interlayer binding energy per Ge/Si atom. When *N = N*(MoS_2_) = 25, the number of sulfur atoms in the supercell, then, *E*_b_ is the interlayer binding energy per MoS_2_. The interlayer binding energies per Ge/Si atom and those per MoS_2_ are presented in Table 1. $E_{{\mathrm{MoS}}_{2}}$ is calculated by using a 5 × 5 unit cell of the MoS_2_ monolayer, and *E*_Ger/Sil_ is calculated by using a 4 × 4 unit cell of the germanene/silicene. The binding energies between the stacking layers of the superlattices, calculated by the PBE-D2 method, are both relatively small, i.e., 0.277 eV/Ge and 0.195 eV/Si for the Ger/MoS_2_ and Sil/MoS_2_ superlattices, respectively (see Table 1). The small interlayer binding energies suggest weak interactions between the germanene/silicene and the MoS_2_ layers. The binding energy also suggests that the interlayer interaction in Ger/MoS_2_ superlattice is slightly stronger than that in the Sil/MoS_2_ one. The interlayer binding energies are 0.354 eV/MoS_2_ and 0.250 eV/MoS_2_ for the Ger/MoS_2_ and Sil/MoS_2_ superlattices, respectively, both are larger than 0.158 eV/MoS_2_ in the graphene/MoS_2_ superlattice [6]. This is an indication that the mixed *sp*^2^-*sp*^3^ hybridization in the buckled germanene and silicene leads to stronger bindings of germanene/silicene with their neighboring MoS_2_ atomic layers, when compared with the pure planar *sp*^2^ bonding in the graphene/MoS_2_ superlattice. In addition, the interlayer bindings become stronger and stronger in the superlattices of graphene/MoS_2_ to silicene/MoS_2_ and then to germanene/MoS_2_ monolayer.

**Figure 2:**
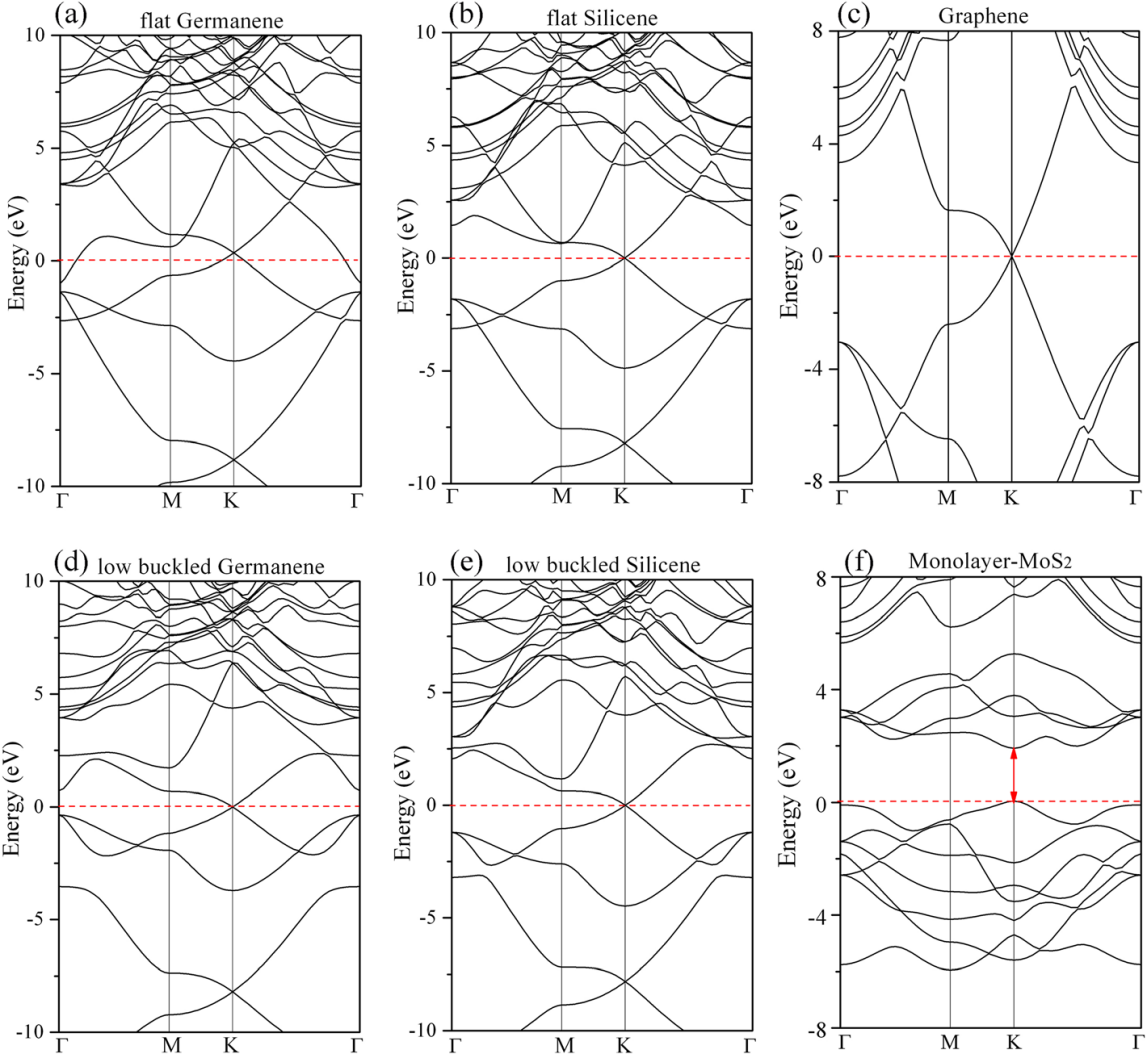
**Band structures of various 2D materials. (a)** Flat germanene, **(b)** flat silicene, **(c)** graphene, **(d)** low-buckled germanene, **(e)** low-buckled silicene, and **(f)** MoS_2_ monolayer.

**Figure 3:**
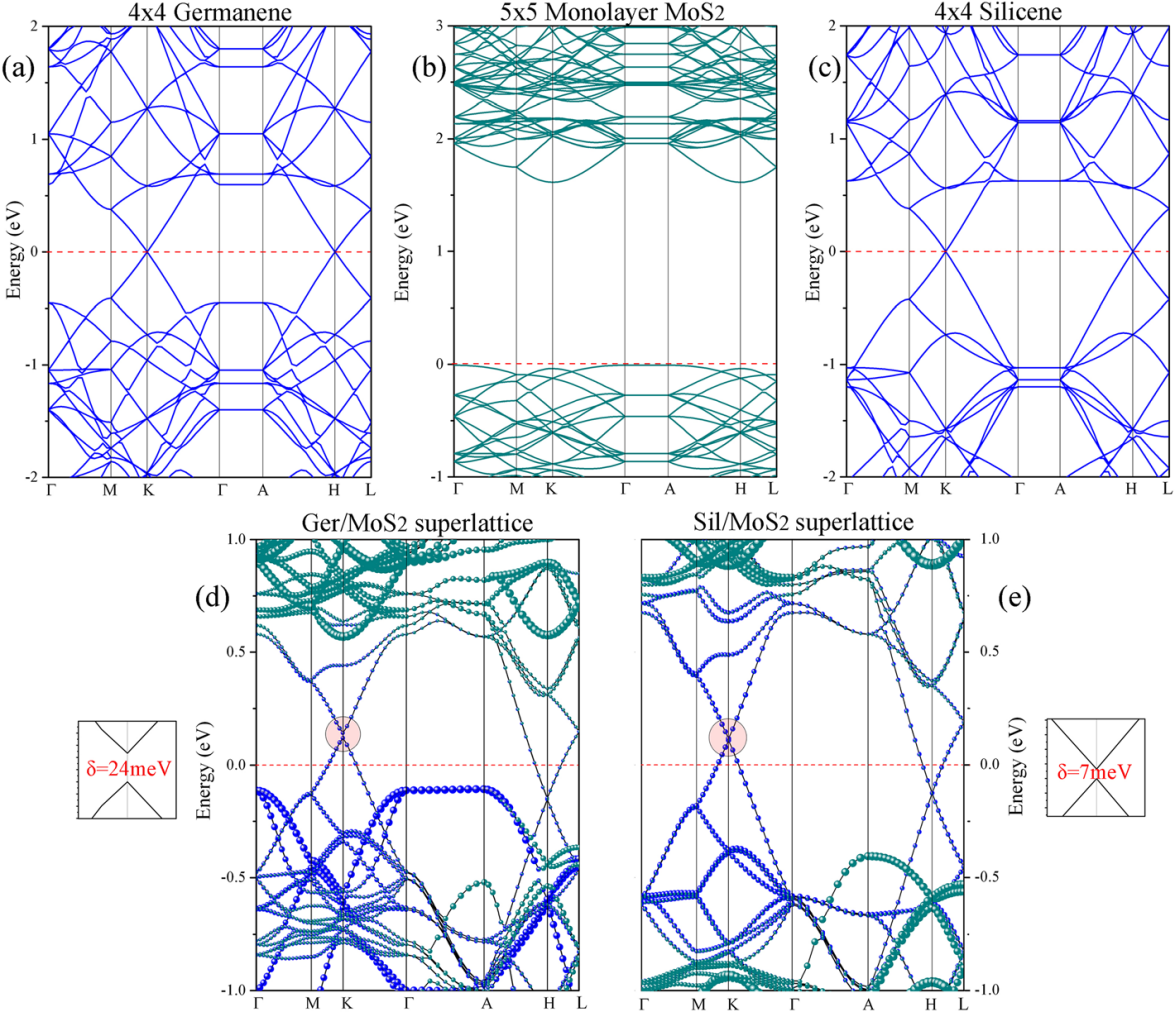
**Band structures of free-standing. (a)** Germanene calculated with a 4 × 4 supercell, **(b)** MoS_2_ monolayer calculated with a 5 × 5 supercell, and **(c)** silicene calculated with a 4 × 4 supercell. **(d,e)** The band structures of Ger/MoS_2_ and Sil/MoS_2_ superlattices, respectively. The contributions from the germanene/silicene and MoS_2_ layers to the band structures of the superlattices are shown with blue and green dots, respectively. The detailed band structures in the vicinity of the opened band gap are inserted. Red dashed lines represent the Fermi level.

Figure 2 shows the band structures of various 2D materials, e.g., the bands of flat germanene/silicene compared with low-buckled germanene/silicene. The band structure of flat silicene is similar to that of low-buckled one. In both kinds of silicene, the systems are semimetal with linear bands around the Dirac point at the *K* point of the Brillouin zone. On the other hand, the band structure of flat germanene is quite different from that of low-buckled one. The flat germanene is metallic, and the Dirac point does not sit at the Fermi level (but above the *E*_F_). The band structure of low-buckled germanene, however, is similar to that of the low-buckled silicene. To help understand the electronic band structures of the superlattices and the contribution of each atomic layer to the band structures, we present in Figure 3 the band structures of Ger/MoS_2_ and Sil/MoS_2_ superlattices, together with those of the independent low-buckled germanene/silicene and MoS_2_ monolayer sheets. The band structures of free-standing buckled germanene/silicene and MoS_2_ sheets (Figure 3a,b,c) are calculated by using 4 × 4 and 5 × 5 supercells, respectively, in order to compare with the band structures of the superlattices directly. The band structures of the Ger/MoS_2_ and Sil/MoS_2_ superlattices are presented in Figure 3d,e, where the contributions of the germanene/silicene and MoS_2_ monolayers to the band structures of the superlattices are shown with blue and green dots (where the size of dots are proportional to the contributions), respectively. In general, the outlines of the band structures of the two superlattices seem to be similar to the ‘rigid sum’ of the bands of each constituent (i.e., the bands of independent germanene/silicene and MoS_2_ sheets), indicating that the couplings between the stacking sheets are relatively weak. However, new important characters in the band structures of the superlattices appear. Both the Ger/MoS_2_ and Sil/MoS_2_ superlattice systems manifest metallic properties, since there are several bands crossing the Fermi level. In fact, in the superlattice systems, the Dirac points of the free-standing germanene/silicene (at the *K* point) move upward slightly above the Fermi level; at the same time, the Dirac points at the *H* point (*H* is above *K* in the *z*-direction in the BZ) move downward slightly below the Fermi level. Such shifts of Dirac points lead to partially occupied bands in the superlattices, also implying charge transfer around *K* point to the *H* point in the BZ. The bands crossing the Fermi level are contributed mainly by the germanene/silicene layers rather than the MoS_2_ sheets in both the Ger/MoS_2_ and Sil/MoS_2_ superlattices, except that small contributions from MoS_2_ sheet are visible around the *H* point. Contributions from the MoS_2_ layers to the electronic states around the Fermi level are more significantly visible in the system of Ger/MoS_2_ than in the Sil/MoS_2_ system. The feature of energy bands suggests that the electronic conduction of the superlattices exists mainly in the *x-y* plane and is almost contributed by the germanene/silicene sheets rather than the MoS_2_ sheets, namely, the superlattices are compounds made with alternate stacking of conductive germanene/silicene layers and nearly insulating MoS_2_ sheets. This is different from the graphene/MoS_2_ superlattice, in which both graphene and MoS_2_ layers can be conductive, resulting from the charge transfer between the graphene and MoS_2_ sheets [6]. Moreover, according to the detailed band structures inserted in the vicinity of Figure 3d,e, we found that small band gaps opened up at the *K* point of the BZ (the Dirac point of the germanene/silicene), which is now above the Fermi level. The gaps that opened for the Ger/MoS_2_ and Sil/MoS_2_ superlattices are 24 and 7 meV, respectively (the sizes of the gaps could be well under-estimated). Since the electronic states around *K* point are almost fully contributed from the germanene/silicene layers, the gaps that opened for the superlattices are due to the interactions between the germanene/silicene layers only. In other words, the formation of the small-sized band gaps at the *K* point is due to the symmetry breaking within the germanene/silicene layers caused by the introduction of the MoS_2_ sheets in the formation of superlattices [43-46].

**Figure 4:**
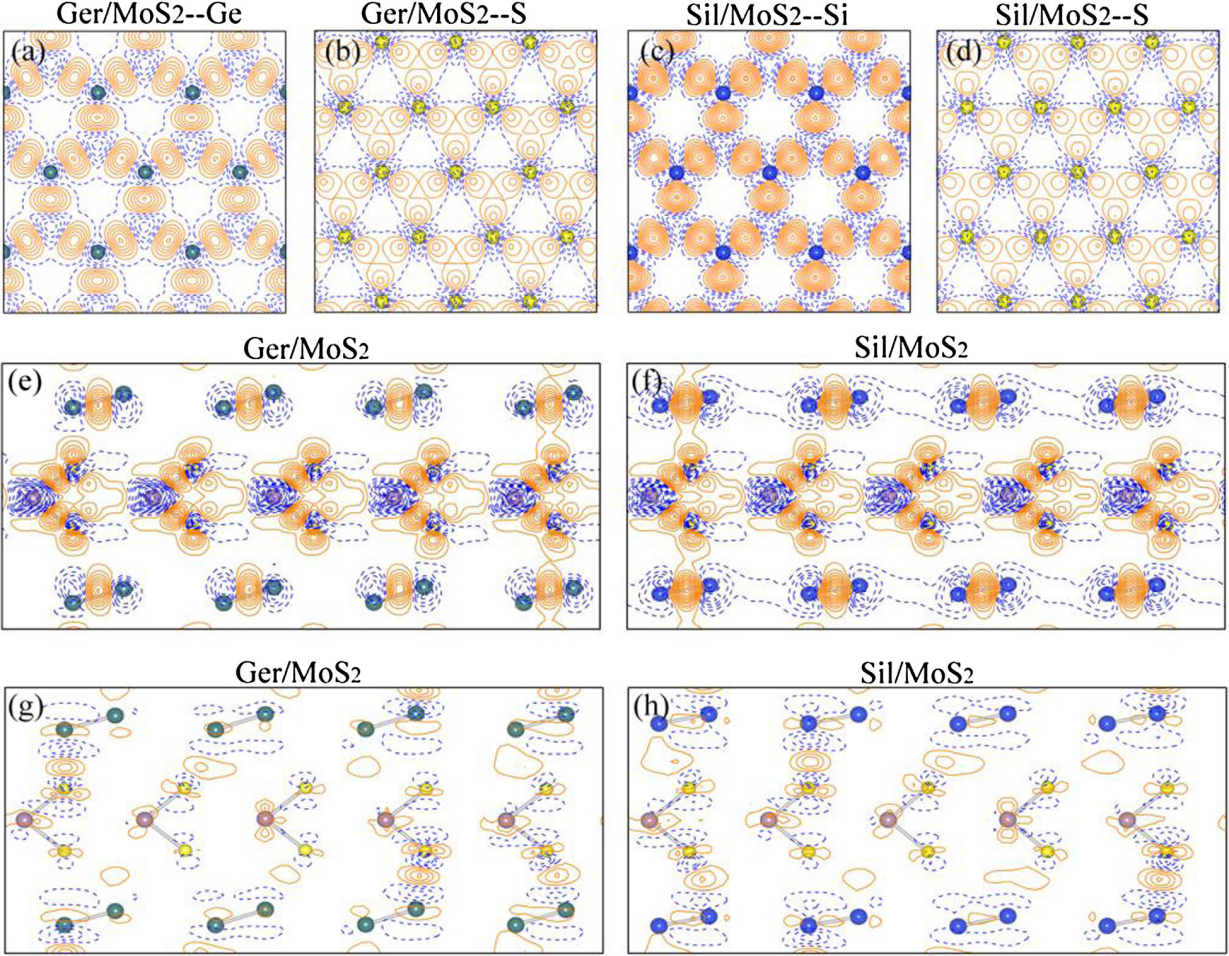
**Contour plots of the deformation charge density (∆*ρ***_**1**_**and ∆*ρ***_**2**_**). (a, b)** ∆*ρ*_1_ on the planes passing through germanene and sulfur layers in the Ger/MoS_2_ superlattice. **(c,d)** ∆*ρ*_1_ on the planes passing through silicene and sulfur layers in the Sil/MoS_2_ system. **(e,f)** ∆*ρ*_1_ on the planes perpendicular to the atomic layers and passing through Mo-S, Ge-Ge, or Si-Si bonds in the superlattices. **(g,h)** Charge density differences (∆*ρ*_2_) of the same planes as those in **(e)** and **(f)**. The green/blue, purple, and yellow balls represent Ge/Si, Mo, and S atoms, respectively. Orange and blue lines correspond to Δ*ρ* > 0 and Δ*ρ* < 0, respectively.

To further explore the bonding nature and the charge transfer in the Ger/MoS_2_ and Sil/MoS_2_ superlattices, the contour plots of the charge density differences (∆*ρ*_1_) on the planes passing through germanene, silicene, and sulfur layers (in the *x*-*y* plane) are shown in Figure 4a,b,c,d. The deformation charge density ∆*ρ*_1_ is defined as $\Delta \rho_{1}\left(\vec{r}\right)=\rho \left(\vec{r}\right)-\sum_{\mu }\rho_{\mathrm{atom}}\left(\vec{r}-{\vec{R}}_{\mu }\right)$, where $\rho \left(\vec{r}\right)$ represents the total charge density of the superlattice and $\sum_{\mu }\rho_{\mathrm{atom}}\left(\vec{r}-{\vec{R}}_{\mu }\right)$ is the superposition of atomic charge densities. The deformation charge density shown in Figure 4a,b,c,d exhibited that the formation of the Ger/MoS_2_ and Sil/MoS_2_ superlattices did not distort significantly the charge densities of germanene, silicene, or sulfur layers, when compared with the deformation charge density in the free-standing germanene, silicene layers, or sulfur layers in the MoS_2_ sheets (not shown). Figure 4e,f shows the contour plots of ∆*ρ*_1_ on the planes perpendicular to the atomic layers and passing through Mo-S, Ge-Ge, or Si-Si bonds in the Ger/MoS_2_ and Sil/MoS_2_ superlattices. As in the case of isolated germanene/silicene or MoS_2_ monolayer (not presented), the atomic bonding within each atomic layer in both the superlattices are mainly covalent bonds. Moreover, shown in Figure 4g,h, we also present the charge density differences (∆*ρ*_2_) of the same planes as in Figure 4e,f. The ∆*ρ*_2_ is defined as $\Delta \rho_{2}\left(\vec{r}\right)=\rho \left(\vec{r}\right)-\rho_{\mathrm{slab}}\left(\mathrm{Ger}/\mathrm{Sil}\right)-\rho_{\mathrm{slab}}\left({\mathrm{MoS}}_{2}\right)$, where $\rho \left(\vec{r}\right)$, *ρ*_slab_(Ger/Sil), and *ρ*_slab_(MoS_2_) are the charge densities of the superlattice, the germanene/silicene, and the MoS_2_ slabs, respectively. In the calculation of *ρ*_slab_(Ger/Sil) and *ρ*_slab_(MoS_2_), we employ the same supercell that is used for the superlattice. For calculating the *ρ*_slab_(Ger/Sil), the MoS_2_ slabs in the superlattice are removed and the charge densities of the germanene/silicene slabs are then calculated including a structure relaxation. For calculating *ρ*_slab_(MoS_2_), the germanene/silicene layers are then removed. Such a ∆*ρ*_2_ can clearly demonstrate the charge transfer between the stacking layers in the superlattices. Figure 4g,h indicates that the charge transfer happened mainly within the germanene/silicene and the MoS_2_ layers (intra-layer transfer), as well as in some parts of the intermediate regions between the germanene/silicene and MoS_2_ layers (inter-layer transfer). This is somewhat different from the graphene/MoS_2_ superlattice, where the charge transfer from the graphene sheet to the intermediate region between the graphene and MoS_2_ layers is much more significantly visible [6]. Such charge redistributions in the Ger/MoS_2_ and Sil/MoS_2_ systems, shown in Figure 4, indicate that the interactions between some parts of the stacking atomic layers are relatively strong, suggesting much more than just the van der Waals interactions between the stacking sheets.

## Conclusions

In summary, the first principles calculations based on density functional theory including van der Waals corrections have been carried out to study the structural and electronic properties of superlattices composed of germanene/silicene and MoS_2_ monolayer. Due to the relatively weak interactions between the stacking layers, the distortions of the geometry of germanene, silicene and MoS_2_ layers in the superlattices are all relatively small. Unlike the free-standing germanene or silicene which is a semimetal and the MoS_2_ monolayer which is a semiconductor, both the Ger/MoS_2_ and Sil/MoS_2_ superlattices exhibit metallic electronic properties. Due to symmetry breaking, small band gaps are opened up at the *K* point of the BZ for both the superlattices. Charge transfer happened mainly within the germanene/silicene and the MoS_2_ layers (intra-layer charge transfer), as well as in some parts of the intermediate regions between the germanene/silicene and MoS_2_ layers (inter-layer charge transfer). Such charge redistributions indicate that the interactions between some parts of the stacking layers are relatively strong, suggesting more than just the van der Waals interactions between the stacking sheets.

## Competing interests

The authors declare that they have no competing interests.

## Authors' contributions

XL carried out the density functional theory simulation, performed the data analysis, and drafted the manuscript. SW and SZ helped discuss the data analysis of the superlattice. ZZ organized the final manuscript. All authors read and approved the final manuscript.
